# Trend of incidence rate of age-related diseases: results from the National Health Insurance Service–National Sample Cohort (NHIS-NSC) database in Korea: a cross- sectional study

**DOI:** 10.1186/s12877-023-04578-7

**Published:** 2023-12-12

**Authors:** In Sun Ryou, Sang Wha Lee, Hanbit Mun, Jae Kwang Lee, SungYoun Chun, Kyunghee Cho

**Affiliations:** 1 Department of Familial Medicine, Ewha Womens University Medical Center, Ewha Womens University School of Medicine, Seoul, Republic of Korea; 2 Department of Family Medicine and Geriatrics, National Health Insurance Service Ilsan Hospital, Goyang-si, Republic of Korea; 3 Department of Research and Analysis, National Health Insurance Service Ilsan Hospital, Goyang-si, Republic of Korea

**Keywords:** Age-related disease, Senescence cell, Burden of disease, Disability-adjusted life-years

## Abstract

**Background:**

This study aimed to identify and select age-related diseases (ARDs) in Korea, which is about to have a super-aged society, and to elucidate patterns in their incidence rates.

**Methods:**

The National Health Insurance Service–National Sample Cohort, comprising 1 million health insurance and medical benefit beneficiaries in Korea from 2002 to 2019, was utilized. We selected 14 diseases with high disease burden and prevalence among Koreans from the 92 diseases defined in the Global Burden of Diseases, Injuries, and Risk Factors Study as ARDs. The annual incidence rate represented the number of patients newly diagnosed with an ARD each year from 2006 to 2019, excluding those with a history of ARD diagnosis from 2002 to 2005. The incidence rate by age was categorized into 10-year units based on age as of 2019. The number of patients with ARDs in each age group was used as the numerator, and the incidence rate for each age group was calculated with the age group as the denominator.

**Results:**

Regarding the annual incidence rates of ARDs from 2006 to 2019, chronic obstructive pulmonary disease, congestive heart failure, and ischemic heart disease decreased annually, whereas dyslipidemia, chronic kidney disease, cataracts, hearing loss, and Parkinson's disease showed a significant increase. Hypertension, diabetes, cerebrovascular disease, osteoporosis, osteoarthritis, and age-related macular degeneration initially displayed a gradual decrease in incidence but exhibited a tendency to increase after 2015. Concerning age-specific incidence rates of ARDs, two types of curves emerged. The first type, characterized by an exponential increase with age, was exemplified by congestive heart failure. The second type, marked by an exponential increase peaking between ages 60 and 80, followed by stability or decrease, was observed in 13 ARDs, excluding congestive heart failure. However, hypertension, ischemic heart disease, cerebrovascular disease, chronic obstructive pulmonary disease, and hearing loss in men belonged to the first type.

**Conclusions:**

From an epidemiological perspective, there are similar characteristics in age-specific ARDs that increase with age, reaching a peak followed by a plateau or decrease in Koreans.

**Supplementary Information:**

The online version contains supplementary material available at 10.1186/s12877-023-04578-7.

## Background

The older population is increasing worldwide [1], and Korea has the fastest aging rate among the major countries in the Organization for Economic Co-operation and Development [2]. According to a report by the National Statistical Office, the rate of aging in Korea increased at an average annual rate of 3.3% from 1970 to 2018, and Korea is expected to become a super-aged society by 2025 [3]. An increase in the older population is a significant cause of increased medical expenses and financial burden on health insurance [4]. For example, in 2019, medical expenses for the older population (age ≥ 65 years) in Korea accounted for 41.6% of the total medical expenses and increased by 9.3% over the past decade. In addition, the annual medical cost per older population individual is 4.91 million KRW (~ 3,600 USD), three times the annual medical cost per non-older population individual [5].

It is essential to pay more attention to age-related diseases (ARDs), as a significant portion of medical expenses and healthcare burden will be concentrated in the older population with the rapid advent of super-aging. ARDs generally refer to diseases that increase in incidence with age, including chronic diseases such as hypertension, diabetes, cardiovascular disease, cerebrovascular disease, Alzheimer disease, Parkinson's disease, age-related macular degeneration, osteoarthritis, osteoporosis, and cancer [6–12]. However, no consistent consensus has defined the disease categories included in ARD. The distinction between normal aging, which occurs naturally with advancing age, and pathological aging remains unclear. As geriatric diseases are a combination of age-related decline in function and disease, the disease categories included in ARD are slightly different depending on the literature [6–12]. Among these, we focused on 92 ARDs classified by defining ARDs as those with exponentially increasing incidence with age, out of a total of 293 causes of disease from the Global Burden of Diseases, Injuries, and Risk Factors Study (GBD) 2017 [13]. The GBD evaluated the burden of each disease of ARDs using the disability-adjusted life-years (DALYs), and ARDs accounted for 51.3% of the total disease burden globally based on the data from the 2017 GBD and the top 10 diseases that had the largest absolute increases in the number of DALYs between 1990 and 2019, including six diseases corresponding to ARD, such as ischemic heart disease, chronic kidney disease, lung cancer, and hearing loss [14].

ARD-related studies in Korea have usually focused on specific diseases such as macular degeneration, hearing loss, and Alzheimer disease [15–17]. In addition, although the Geriatrics Fact Sheet and Burdens of Disease of the Older Population have been published using a Korean national cohort [4, 18], no studies have analyzed ARDs or their incidence according to year and age. Therefore, we aimed to identify and select ARDs in terms of disease burden to provide information for the management of the older population in Korea and to analyze their characteristics by evaluating the incidence rate of each disease.

## Methods

### Definition of ARD

We defined ARD as a disease with a burden that increases with age and an incidence rate that increases exponentially with age. To determine the disease group belonging to ARDs, we evaluated a list of 293 causes of disease from the GBD using the two-step method described by Chang et al., which excludes diseases that do not have a positive correlation between the incidence rate and age as well as diseases whose incidence does not increase exponentially with age; finally, a total of 92 ARDs were identified [13]. In addition, in 2020, the Ministry of Health and Welfare confirmed the results of a survey on older individuals to examine the causes of diseases with high prevalence in the older population in Korea [18]. In a study on the burden of disease in Koreans, the top five specific causes of DALYs by age were examined, and diseases with high incidence rates were identified [19]. Finally, 14 diseases were selected as ARDs after consultation with researchers and clinicians. Cancer, a representative ARD, was excluded from this study.

### Data sources

This study used data from the National Health Insurance Service–National Sample Cohort (NHIS-NSC). This database included data from a sample of 1 million individuals who maintained health insurance and were beneficiaries of medical benefits in Korea for 1 year in 2006 and were followed up from 2002 to 2019. The NHIS-NSC data include sociodemographic information, outpatient and inpatient records, pharmacy claims, health examination results, and data on deaths collected by the National Statistical Office, such that the date and cause of death can be determined. The obtained information was then extracted, summarized, processed, and anonymized to ensure that the subjects cannot be identified [20]. The authors received approval from the Ethics Committee of the National Health Insurance Corporation to use this data. The research was conducted after receiving approval (IRB number: NHIS-2021–1-459) from the Ethics Committee of the National Health Insurance Ilsan Hospital.

### Incidence rate of ARDs

The diagnostic codes for the 14 diseases classified as ARDs were extracted from individuals with records of these diseases in the NHIS-NSC data. The diagnostic codes used were based on the 8th revised Korea Standard Disease Classification, and they are summarized in Additional file 1.

The annual incidence rate was derived from patients with new occurrences of each ARD each year for a total of 14 years (from 2006 to 2019), excluding patients with a history of being diagnosed with each ARD from 2002 to 2005. The incidence rate for 2006 to 2019 was calculated using the number of patients with ARDs as the numerator and the number of people who did not develop each ARD in the previous year as the denominator. The incidence rate by age was divided into 10-year increments by age as of 2019. To calculate the incidence rate of each ARD by age group, the number of patients with ARDs in each age group was used as the numerator, and the number of people for each age in 2019 was used as the denominator. SAS version 9.4 (SAS Institute, Cary, NC, USA) was used for data pre-processing and incidence rate calculation.

## Results

### Disease categories of ARD

The 14 ARDs included hypertension, diabetes, dyslipidemia, cerebrovascular disease, ischemic heart disease, osteoporosis, osteoarthritis, chronic obstructive pulmonary disease, congestive heart failure, chronic kidney disease, cataracts, age-related macular degeneration, hearing loss, and Parkinson's disease.

### Incidence rates of ARDs per year

**Fig. 1:**
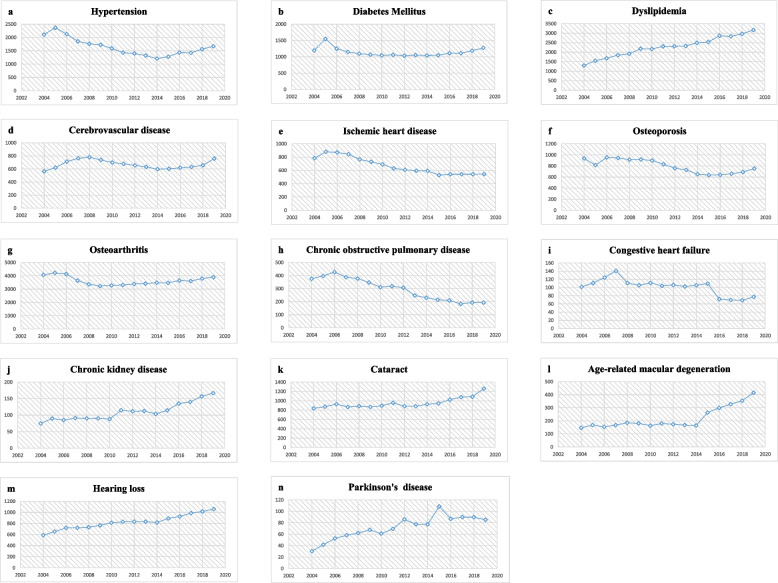
Incidence rates of age-related diseases by year per 100,000 persons

Examination of the annual incidence rate per 100,000 people by disease showed that hypertension gradually decreased from 2006, rebounding from its lowest incidence rate in 2014 and demonstrating a gradual increase in the incidence rate (Fig. 1). Meanwhile, the incidence rate of diabetes gradually decreased and experienced a slight increase from 2018, but the changes did not appear to be statistically significant. The incidence rate of dyslipidemia continued to rise. The incidence rate of cerebrovascular disease increased until 2008, then decreased, and started to gradually increase again from 2016, while that of ischemic heart disease showed a gradual decrease. Osteoporosis also showed a steady decline but showed a tendency to increase after 2016. The incidence of osteoarthritis declined until 2009, followed by a gradual increase. The incidence rate of chronic obstructive pulmonary disease gradually decreased. In the case of congestive heart failure, the incidence rate peaked in 2007, remained relatively stable from 2008 to 2015, started to decrease after 2015, and has since maintained a similar pattern, whereas chronic kidney disease showed a moderate incidence but increased after 2015. In the case of cataracts, the incidence rate continued to increase gradually, and the incidence rate of age-related macular degeneration, which remained stable, increased rapidly after 2014, whereas the incidence rate of hearing loss increased gradually over the years. In the case of Parkinson’s disease, the incidence rate itself was not high and varied by year but showed an overall increasing trend (Table 1).

**Table 1:** Incidence rate of age-related disease by year

		**2006**	**2007**	**2008**	**2009**	**2010**	**2011**	**2012**	**2013**	**2014**	**2015**	**2016**	**2017**	**2018**	**2019**
**HTN**	Total number of subjects (n)	920,747	908,912	897,268	887,432	878,180	869,934	865,101	859,197	854,180	852,055	849,232	842,585	836,784	829,077
	number of occurrences(n)	19,581	16,873	15,810	15,296	13,987	12,435	12,133	11,363	10,310	10,896	12,205	11,987	13,082	13,867
	Incidence rate (%)	2.13	1.86	1.76	1.72	1.59	1.43	1.40	1.32	1.21	1.28	1.44	1.42	1.56	1.67
**DM**	Total number of subjects (n)	967,764	965,139	958,460	952,784	948,377	944,360	940,484	936,565	932,507	929,035	925,146	919,108	913,200	906,226
	number of occurrences(n)	12,105	11,111	10,513	10,202	9920	10,018	9729	9894	9708	9757	10,291	10,213	10,848	11,554
	Incidence rate (%)	1.25	1.15	1.10	1.07	1.05	1.06	1.03	1.06	1.04	1.05	1.11	1.11	1.19	1.27
**DL**	Total number of subjects (n)	983,471	978,946	965,438	953,561	937,722	923,481	907,638	891,399	877,282	861,210	846,926	825,942	806,693	786,955
	number of occurrences(n)	16,536	18,073	18,490	20,783	20,335	21,296	20,986	20,710	21,854	21,815	24,279	23,388	23,919	24,939
	Incidence rate (%)	1.68	1.85	1.92	2.18	2.17	2.31	2.31	2.32	2.49	2.53	2.87	2.83	2.97	3.17
**CVD**	Total number of subjects (n)	1,005,132	1,008,944	1,006,256	1,003,050	1,001,241	999,757	999,073	997,739	996,921	996,995	996,324	994,413	991,636	988,869
	number of occurrences(n)	7171	7707	7850	7402	6994	6789	6536	6308	5951	5995	6169	6285	6515	7491
	Incidence rate (%)	0.71	0.76	0.78	0.74	0.70	0.68	0.65	0.63	0.60	0.60	0.62	0.63	0.66	0.76
**IHD**	Total number of subjects (n)	993,762	995,291	990,594	987,171	985,102	982,905	982,193	980,907	980,269	979,286	978,704	976,928	974,277	971,133
	number of occurrences(n)	8653	8393	7577	7200	6805	6214	5974	5842	5809	5184	5305	5297	5286	5285
	Incidence rate (%)	0.87	0.84	0.76	0.73	0.69	0.63	0.61	0.60	0.59	0.53	0.54	0.54	0.54	0.54
**Osteoporosis**	Total number of subjects (n)	991,039	991,929	986,442	981,770	977,914	973,564	970,424	967,579	965,015	964,098	962,601	960,280	957,155	953,415
	number of occurrences(n)	9456	9357	9017	9012	8771	8102	7372	7060	6292	6135	6164	6337	6617	7173
	Incidence rate (%)	0.95	0.94	0.91	0.92	0.90	0.83	0.76	0.73	0.65	0.64	0.64	0.66	0.69	0.75
**OA**	Total number of subjects (n)	874,646	843,531	815,805	792,941	774,316	755,913	738,821	720,612	703,395	685,954	670,024	650,631	633,224	614,019
	number of occurrences(n)	36,161	30,698	27,480	25,690	25,370	25,017	25,039	24,641	24,501	23,805	24,460	23,458	24,003	23,917
	Incidence rate (%)	4.13	3.64	3.37	3.24	3.28	3.31	3.39	3.42	3.48	3.47	3.65	3.61	3.79	3.90
**COPD**	Total number of subjects (n)	1,008,975	1,014,715	1,014,958	1,015,170	1,016,477	1,018,279	1,020,378	1,021,068	1,023,158	1,025,554	1,027,235	1,027,772	1,028,068	1,027,514
	number of occurrences(n)	4317	3930	3825	3527	3164	3244	3133	2523	2360	2202	2150	1887	1987	1990
	Incidence rate (%)	0.43	0.39	0.38	0.35	0.31	0.32	0.31	0.25	0.23	0.21	0.21	0.18	0.19	0.19
**CHF**	Total number of subjects (n)	1,017,969	1,027,330	1,029,461	1,032,154	1,035,853	1,038,972	1,042,775	1,045,416	1,048,484	1,051,746	1,053,372	1,054,804	1,055,281	1,055,277
	number of occurrences(n)	1267	1448	1142	1092	1152	1084	1108	1072	1109	1155	756	735	725	815
	Incidence rate (%)	0.12	0.14	0.11	0.11	0.11	0.10	0.11	0.10	0.11	0.11	0.07	0.07	0.07	0.08
**CKD**	Total number of subjects (n)	1,018,323	1,027,969	1,030,743	1,033,510	1,037,106	1,040,588	1,044,006	1,046,462	1,049,144	1,052,300	1,054,361	1,055,095	1,054,976	1,054,008
	number of occurrences(n)	866	935	926	934	914	1194	1163	1179	1088	1203	1424	1476	1655	1755
	Incidence rate (%)	0.09	0.09	0.09	0.09	0.09	0.11	0.11	0.11	0.10	0.11	0.14	0.14	0.16	0.17
**Cataract**	Total number of subjects (n)	993,521	994,138	990,302	986,076	983,524	980,933	977,087	973,825	971,544	968,796	965,773	960,740	954,191	948,574
	number of occurrences(n)	9224	8619	8767	8555	8783	9369	8640	8586	9002	9128	9886	10,348	10,384	11,952
	Incidence rate (%)	0.93	0.87	0.89	0.87	0.89	0.96	0.88	0.88	0.93	0.94	1.02	1.08	1.09	1.26
**AMD**	Total number of subjects (n)	1,017,115	1,026,146	1,028,181	1,029,794	1,032,231	1,034,751	1,037,393	1,039,063	1,041,194	1,044,611	1,045,297	1,044,579	1,042,582	1,040,138
	number of occurrences(n)	1566	1716	1893	1863	1681	1857	1802	1739	1707	2752	3125	3418	3688	4322
	Incidence rate (%)	0.15	0.17	0.18	0.18	0.16	0.18	0.17	0.17	0.16	0.26	0.30	0.33	0.35	0.42
**Hearing loss**	Total number of subjects (n)	1,005,248	1,008,436	1,005,137	1,001,983	999,476	995,900	992,691	988,475	984,485	981,825	977,258	971,463	964,014	955,725
	number of occurrences(n)	7222	7256	7354	7667	8108	8262	8251	8226	8035	8742	9039	9563	9792	10,110
	Incidence rate (%)	0.72	0.72	0.73	0.77	0.81	0.83	0.83	0.83	0.82	0.89	0.92	0.98	1.02	1.06
**PD**	Total number of subjects (n)	1,020,231	1,030,203	1,033,239	1,036,197	1,039,875	1,043,351	1,047,355	1,049,913	1,052,973	1,056,531	1,058,105	1,059,204	1,059,267	1,058,699
	number of occurrences(n)	536	598	640	700	633	722	899	812	813	1146	918	951	949	902
	Incidence rate (%)	0.05	0.06	0.06	0.07	0.06	0.07	0.09	0.08	0.08	0.11	0.09	0.09	0.09	0.09

### Incidence rates of ARDs per age group

**Fig. 2:**
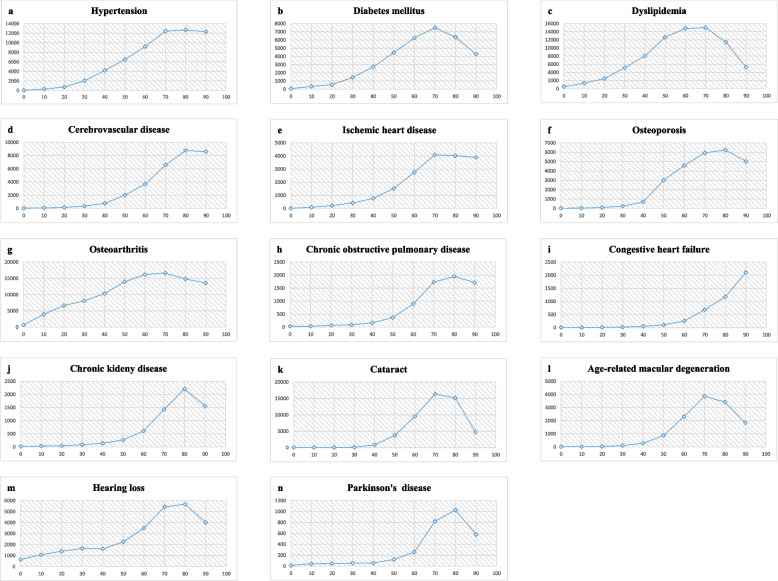
Incidence rates of age-related diseases by age group per 100,000 persons

The age-specific incidence rate of each disease generally increases with age but can be broadly categorized into two types. The first type was characterized by an exponential increase in the incidence rate with age, with congestive heart failure corresponding to this pattern (Table 2, Fig. 2). When analyzed separately by sex, hypertension, ischemic heart disease, cerebrovascular disease, chronic obstructive pulmonary disease, and hearing loss also showed an increase in men (Additional files 2 and 3). The second type was characterized by an exponential increase in the incidence rate with age, reaching a peak between the ages of 60 and 80 years, after which the incidence rate either remained stable or decreased (Table 2, Fig. 2). In this study, 13 ARDs, excluding congestive heart failure, followed this pattern. When differentiated by sex, all diseases, except congestive heart failure and the five previously mentioned diseases, fell into the second category (Additional files 4–7).

**Table 2:** Incidence rate of age-related disease by age group

		**0**	**10**	**20**	**30**	**40**	**50**	**60**	**70**	**80**	**90**	**Total**
**HTN**	Total number of subjects (n)	86,786	102,557	138,776	137,285	148,289	123,859	63,756	21,005	5951	813	829,077
	number of occurrences(n)	29	288	1034	2798	6190	8054	5866	2608	754	100	27,721
	Incidence rate (%)	0.03	0.28	0.75	2.04	4.17	6.50	9.20	12.42	12.67	12.30	3.34
**DM**	Total number of subjects (n)	86,824	102,140	138,534	138,725	154,899	142,051	85,267	38,123	16,702	2961	906,226
	number of occurrences(n)	59	344	766	1998	4214	6342	5334	2858	1058	126	23,099
	Incidence rate (%)	0.07	0.34	0.55	1.44	2.72	4.46	6.26	7.50	6.33	4.26	2.55
**DL**	Total number of subjects (n)	86,722	100,550	133,628	126,999	132,861	108,035	55,491	26,036	13,763	2870	786,955
	number of occurrences(n)	420	1338	3324	6508	10,708	13,680	8218	3910	1576	152	49,834
	Incidence rate (%)	0.48	1.33	2.49	5.12	8.06	12.66	14.81	15.02	11.45	5.30	6.33
**CVD**	Total number of subjects (n)	86,789	102,686	140,376	143,073	166,393	164,036	109,348	52,871	20,359	2938	988,869
	number of occurrences(n)	34.00	74.00	214.00	512.00	1284.00	3300.00	4044.00	3472.00	1786.00	252.00	14,972.00
	Incidence rate (%)	0.04	0.07	0.15	0.36	0.77	2.01	3.70	6.57	8.77	8.58	1.51
**IHD**	Total number of subjects (n)	86,849	102,752	140,005	141,605	163,133	157,831	102,704	50,832	21,922	3500	971,133
	number of occurrences(n)	6	88	310	598	1236	2402	2832	2078	882	136	10,568
	Incidence rate (%)	0.01	0.09	0.22	0.42	0.76	1.52	2.76	4.09	4.02	3.89	1.09
**Osteoporosis**	Total number of subjects (n)	86,886	102,914	140,899	143,432	166,771	158,853	95,768	40,950	14,753	2189	953,415
	number of occurrences(n)	22	36	132	344	1158	4796	4394	2432	920	110	14,344
	Incidence rate (%)	0.03	0.03	0.09	0.24	0.69	3.02	4.59	5.94	6.24	5.03	1.50
**OA**	Total number of subjects (n)	85,991	95,613	113,152	100,460	101,660	72,809	31,500	9775	2690	369	614,019
	number of occurrences(n)	591	3754	7514	8118	10,484	10,144	5086	1626	398	50	47,765
	Incidence rate (%)	0.69	3.93	6.64	8.08	10.31	13.93	16.15	16.63	14.80	13.55	7.78
**COPD**	Total number of subjects (n)	86,729	102,496	140,333	143,366	168,024	170,598	120,195	64,017	27,664	4092	1,027,514
	number of occurrences(n)	29	34	96	128	276	628	1070	1106	538	70	3975
	Incidence rate (%)	0.03	0.03	0.07	0.09	0.16	0.37	0.89	1.73	1.94	1.71	0.39
**CHF**	Total number of subjects (n)	86,867	102,927	141,141	144,629	170,243	174,407	126,450	71,297	32,467	4849	1,055,277
	number of occurrences(n)	5	4	20	34	84	184	322	488	382	102	1625
	Incidence rate (%)	0.01	0.00	0.01	0.02	0.05	0.11	0.25	0.68	1.18	2.10	0.15
**CKD**	Total number of subjects (n)	86,888	102,943	141,048	144,390	169,709	173,616	125,802	71,189	33,285	5138	1,054,008
	number of occurrences(n)	14	32	58	120	230	460	762	1016	734	80	3506
	Incidence rate (%)	0.02	0.03	0.04	0.08	0.14	0.26	0.61	1.43	2.21	1.56	0.33
**Cataract**	Total number of subjects (n)	86,871	102,710	140,597	143,975	168,059	164,570	99,938	31,901	8558	1395	948,574
	number of occurrences(n)	14	28	74	202	1384	6106	9510	5218	1298	66	23,900
	Incidence rate (%)	0.02	0.03	0.05	0.14	0.82	3.71	9.52	16.36	15.17	4.73	2.52
**AMD**	Total number of subjects (n)	86,890	102,866	140,678	144,190	169,418	172,318	122,836	66,449	29,902	4591	1,040,138
	number of occurrences(n)	4	12	34	142	466	1490	2826	2566	1020	84	8644
	Incidence rate (%)	0.00	0.01	0.02	0.10	0.28	0.86	2.30	3.86	3.41	1.83	0.83
**Hearing loss**	Total number of subjects (n)	85,293	97,922	131,429	133,982	156,811	157,466	108,724	56,090	24,362	3646	955,725
	number of occurrences(n)	544	1040	1838	2208	2514	3550	3802	3038	1380	146	20,060
	Incidence rate (%)	0.64	1.06	1.40	1.65	1.60	2.25	3.50	5.42	5.66	4.00	2.10
**PD**	Total number of subjects (n)	86,898	102,913	140,967	144,459	170,133	174,643	127,450	72,516	33,525	5195	1,058,699
	number of occurrences(n)	10	44	66	78	96	212	330	594	344	30	1804
	Incidence rate (%)	0.01	0.04	0.05	0.05	0.06	0.12	0.26	0.82	1.03	0.58	0.17

## Discussion

In this study, we selected 14 diseases as ARDs, based on their high prevalence in older Koreans and their incidence rates, which increase with age. Accordingly, there was a trend toward decreasing annual incidence rates of chronic obstructive pulmonary disease, congestive heart failure, and ischemic heart disease from 2006 to 2019. In contrast, dyslipidemia, chronic kidney disease, cataracts, hearing loss, and Parkinson’s disease showed increased incidence rates during the same period. The incidence rates of hypertension, diabetes, cerebrovascular disease, osteoporosis, osteoarthritis, and age-related macular degeneration, which had been gradually decreasing, showed a tendency to increase after 2015.

Our main finding was that there are two different types of disease incidence rate curves when examining diseases according to age in 10-year increments. The first type included diseases for which the incidence rate increased exponentially until the oldest age, represented solely by congestive heart failure. However, when analyzed by sex, the incidence rates of hypertension, ischemic heart disease, cerebrovascular disease, chronic obstructive pulmonary disease, and hearing loss in men continued to increase until the oldest age. The other type includes diseases for which the incidence rate increased exponentially and peaked between the ages of 60 and 80 years, after which it plateaued or declined. The majority of diseases fell under this category. Several epidemiological studies have investigated ARDs and their incidence rates. Using a primary database from England, researchers grouped 278 diseases into nine clusters based on the median age of disease onset [21]. They assumed that four clusters containing 207 diseases were related to ARD, showing an increase in incidence rates with age. Although the median age of disease onset decreased from one cluster to another, they consistently exhibited an exponential increase in incidence rates. These clusters included dementia (cluster 1; onset age, 82 years), age-related macular degeneration and heart failure (cluster 2; onset age, 77 years), Parkinson’s disease (cluster 3; onset age, 69 years), and hypertension and osteoarthritis (cluster 4; onset age, 57 years). Another study identified four groups of 92 non-communicable diseases using the 2019 GBD database [22]. They showed that group A diseases had an exponential increase in incidence rates with age; group B diseases had an exponential increase in incidence rates that typically peaked in late life and then declined or plateaued at the oldest ages; groups C and D diseases had an onset in early life with stable or decreased incidence rates in old age. Furthermore, another study concluded that seven diseases were selected as ARD because their doubling time of 8-year Gompertz mortality was the same [23]. In other words, various diseases presumed to belong to ARDs exhibit the characteristic of being grouped based on age-specific incidence rates. This similarity suggests that the general biological aging process dominates the pathogenesis of various diseases, which can be explained by the accumulation of senescent cells and differences in individual susceptibility to diseases [24].

Senescent cells stop dividing in response to various stressors and accumulate in the body with age. They secrete senescence-associated secretory profiles (SASP) to induce inflammation or to reproduce normal cells [25]. According to a study on the relationship between age and senescent cells, the turnover of senescent cells produced and eliminated occurs rapidly during young age; however, with increasing age, the turnover slows, especially the rate of elimination [26], and this was used to develop a statistical probability model for the generation and removal of senescent cells. This is called the saturated-removal (SR) model, and it is confirmed that the accumulation of senescent cells occurs because the generation of senescent cells increases with various stressors as one ages, while the self-removal rate decreases [27]. Nevertheless, because the number of senescent cells differs among individuals, the rate of senescent cell removal varies. Assuming that death occurs when senescent cells exceed the threshold, the SR model can explain the distribution of death time [26, 27]. As aging cells are associated with several ARDs, if ARDs occur when they exceed a specific disease threshold, aging cells secrete SASPs that affect the physiological parameters related to the occurrence of certain diseases, causing the disease to exceed the threshold. Therefore, as the number of aging cells increases exponentially with age, the disease increases exponentially with age [24].

However, while previous studies considered that ARD continues to increase in incidence rate with age, our study showed that most of the ARDs we selected exhibited exponential increases, followed by a peak and a subsequent decrease or plateau. Other studies have also shown that diseases belonging to ARD tend to increase approximately exponentially with age and then decrease in very old age (beyond the peak age), similar to the findings of our study [23, 28]. The decrease in the incidence rate at a very old age can be explained by differences in individual susceptibility to specific diseases [29, 30]. Each population has a different susceptibility to diseases owing to differences in genetic or environmental factors; therefore, the risk of developing a disease may vary. Thus, ARD would occur in individuals with a low threshold for each disease, but it would not occur during normal aging in a population with a high disease threshold. However, most very old people with a low threshold for a disease would have already been afflicted with the disease, and most of the remaining people would have a high threshold for the disease; therefore, the probability of developing a new disease is relatively low, resulting in a decrease in the incidence.

A sub-analysis was performed by dividing the incidence rate of age-specific ARDs by sex. In general, the graph curve of the incidence rate was similar between sexes. However, in the case of hypertension, ischemic heart disease, cerebrovascular disease, chronic obstructive pulmonary disease, and hearing loss in men, unlike in women or all sexes, the incidence rate tends to increase exponentially with age. For vascular diseases, such as hypertension, ischemic heart disease or cerebrovascular disease, the number of disease occurrences continues to increase in men, peaking at 60 years, and then suddenly decreasing at 80 or 90 years. In contrast, in women, it peaks at 60 years and then gradually decreases thereafter. Previous research has shown that, owing to the protective effect of estrogen, the onset of vascular disease occurs later in life, and the mortality rate usually increases after the age of 55 years in women. However, disease onset occurs relatively earlier in men than in women, and the mortality rate is relatively higher in men than in women. Thus, the difference in mortality rates between women and men decreases as they get older [30]. However, our study did not observe a rapid increase in the number of postmenopausal vascular disease events in women. In addition, because women have a longer life expectancy than men, the remaining female population is relatively large. Thus, the incidence rate in patients older than 80 years decreased, even though the number of vascular disease events did not decrease steeply. In contrast, the incidence rate of vascular disease remains high due to the small number of men surviving beyond the age of 80 years, even though the number of cases in men suddenly decreased after the age of 80 years. This phenomenon could be explained by the hypothesis that there is a cohort of healthy survivors with delayed mortality or a cohort of frail individuals with earlier mortality [31]. In the case of chronic obstructive pulmonary disease, one study reported that female patients with chronic obstructive pulmonary disease were younger, had relatively fewer smokers, and had better lung function; however, the incidence rate of dyspnea was higher, and the chronic obstructive pulmonary disease survival rate in women was higher than that in men [32]. Although smoking status and pulmonary function test results were not confirmed in this study, our study showed that the number of cases among men was almost twice as high as that among women, and the survival rate of women was higher than that of men. Unlike men, the number of survivors among women is greater, and the incidence rate among women older than 80 years has decreased. Lastly, in the case of hearing loss, similar to other diseases, the incidence rate is believed to be higher because of the small number of men surviving beyond 80 years.

This study has some limitations. First, since the collected claims data were used for management purposes for insurance claims and refunds, information such as diagnostic codes might be inaccurate, possibly affecting the incidence rate; second, because of this, the actual disease may have been underestimated or overestimated compared with the number of occurrences. Finally, because the disease category of ARD was selected through an expert meeting based on ARDs proposed by Chang et al. [13] and published data, the DALY on the exponential increase in the incidence rate was calculated and not based on ARD.

Nevertheless, this study is meaningful as it represents a large-scale investigation based on a representative sample cohort of one million Koreans. To the best of our knowledge, it is the first research to explore the incidence rates of ARDs across the age spectrum, from 0 to 90 years, within the Korean population, demonstrating the presence of two distinct types of incidence rate curves for age-specific ARDs.

## Conclusion

Our findings showed that the incidence rate of most diseases belonging to ARDs increased exponentially with age and exhibited a consistent pattern of peaking between the ages of 60 and 80 years, followed by a plateau or decrease. However, there were slight differences based on sex among Koreans. Understanding the general characteristics of ARDs and their disease burden could help develop public health policies for healthy aging.

### Supplementary Information

**Additional file 1: Supplementary Table 1.** Diagnostic code of Age-related disease.

**Additional file 2: Supplementary Table 2.** Incidence rate of age-related diseases of Male by year.

**Additional file 3: Supplementary Table 3.** Incidence rate of age-related diseases of Female by year.

**Additional file 4: Supplementary Table 4**. Incidence rate of age-related diseases of Male by age group.

**Additional file 5: Supplementary Table 5.** Incidence rate of age-related diseases of Female by age group.

**Additional file 6: Supplementary Figure 1.** Incidence rate of age-related diseases of Male by age group per 100,000 persons.

**Additional file 7: Supplementary Figure 2.** Incidence rate of age-related diseases of Male by age group per 100,000 persons.

## Data Availability

The data cannot be shared publicly because health information data that are collected, managed, and maintained by the National Health Insurance Corporation must be modified as requested for policy and academic research; however, it can be requested from the corresponding author if there is a reasonable request.

## Abbreviations

ARDs: Age-related diseases
DALYs: Disability-adjusted life-years
GBD: Global Burden of Diseases, Injuries, and Risk Factors Study
NHIS-NSC: National Health Insurance Service–National Sample Cohort
SASP: Senescence-associated secretory profiles
SR: Saturated-removal
