# Supplementary material for: Trend of incidence rate of age-related diseases: results from the National Health Insurance Service–National Sample Cohort (NHIS-NSC) database in Korea: a cross- sectional study
Source: BMC Geriatr. 2023 Dec 12;23:840. doi: 10.1186/s12877-023-04578-7 (PMC10714524; doi:10.1186/s12877-023-04578-7)
Supplement: Supplementary file 1 — Additional file 1: Supplementary Table 1. Diagnostic code of Age-related disease. [file 12877_2023_4578_MOESM1_ESM.pdf]

Supplementary Table 1. Diagnostic code of Age-related disease

Disease	KCD-8 codes
Hypertension	I10 ~ I13
Diabetes mellitus	E10 ~ E14
Dyslipidemia	E78.0 ~ E78.5
Cerebrovascular disease	I60 ~ I69
Ischemic heart disease	I20 ~ I25
Osteoporosis	M80, M81
Osteoarthritis	M15 ~ M19
Chronic obstructive pulmonary disease	J44
Congestive heart failure	I50
Chronic kidney disease	N18, I12
Cataract	H25, H26
Age related macular degeneration	H35.30, H35.31
Hearing loss	H90, H91
Parkinson's disease	G20, G21
