# Supplementary material for: Trend of incidence rate of age-related diseases: results from the National Health Insurance Service–National Sample Cohort (NHIS-NSC) database in Korea: a cross- sectional study
Source: BMC Geriatr. 2023 Dec 12;23:840. doi: 10.1186/s12877-023-04578-7 (PMC10714524; doi:10.1186/s12877-023-04578-7)
Supplement: Supplementary file 2 — Additional file 2: Supplementary Table 2. Incidence rate of age-related diseases of Male by year. [file 12877_2023_4578_MOESM2_ESM.pdf]

Supplementary Table 2. Incidence rate of age-related diseases of Male by year

		2006	2007	2008	2009	2010	2011	2012	2013	2014	2015	2016	2017	2018	2019
HTN	Total number of subjects (n)	2004	2005	2006	2007	2008	2009	2010	2011	2012	2013	2014	2015	2016	2017
	number of occurrences(n)	477,020	473,657	467,230	461,941	455,585	450,324	445,234	440,586	437,404	433,525	430,053	428,551	426,110	421,995
	Incidence rate (%)	9,862	10,685	9,732	8,800	8,291	8,105	7,542	6,927	6,772	6,398	5,742	6,285	6,875	6,896
DM	Total number of subjects (n)	2.07	2.26	2.08	1.91	1.82	1.80	1.69	1.57	1.55	1.48	1.34	1.47	1.61	1.63
	number of occurrences(n)	487,727	489,123	485,341	484,491	481,015	478,338	476,252	474,135	472,163	470,049	467,976	466,274	464,054	460,848
	Incidence rate (%)	5,871	7,406	5,890	5,543	5,178	5,073	5,003	5,048	4,886	4,987	4,940	5,118	5,369	5,411
DL	Total number of subjects (n)	1.20	1.51	1.21	1.14	1.08	1.06	1.05	1.06	1.03	1.06	1.06	1.10	1.16	1.17
	number of occurrences(n)	494,922	495,515	494,067	492,752	486,195	480,818	473,500	467,144	459,738	451,752	445,255	437,543	430,459	420,142
	Incidence rate (%)	6,130	7,147	7,635	8,547	8,623	9,731	9,494	10,173	10,110	9,884	10,659	10,795	12,046	11,753
CVD	Total number of subjects (n)	1.24	1.44	1.55	1.73	1.77	2.02	2.01	2.18	2.20	2.19	2.39	2.47	2.80	2.80
	number of occurrences(n)	498,306	501,622	504,486	506,920	505,954	504,930	504,372	503,931	504,012	503,290	502,830	502,945	502,732	501,640
	Incidence rate (%)	2,542	2,672	3,124	3,323	3,396	3,285	3,100	3,034	3,096	2,995	2,760	2,811	2,972	2,919
IHD	Total number of subjects (n)	0.51	0.53	0.62	0.66	0.67	0.65	0.61	0.60	0.61	0.60	0.55	0.56	0.59	0.58
	number of occurrences(n)	495,355	497,720	498,853	499,894	497,661	496,052	495,054	493,893	493,181	492,355	491,797	491,227	490,612	489,529
	Incidence rate (%)	3,702	4,065	4,172	4,018	3,691	3,552	3,432	3,246	3,010	3,015	2,989	2,782	2,804	2,908
Osteoporosis	Total number of subjects (n)	0.75	0.82	0.84	0.80	0.74	0.72	0.69	0.66	0.61	0.61	0.61	0.57	0.57	0.59
	number of occurrences(n)	500,576	505,662	510,239	514,677	515,154	515,838	516,810	517,318	517,943	518,466	519,155	520,046	520,603	520,572
	Incidence rate (%)	709	776	1,095	1,266	1,299	1,435	1,534	1,509	1,273	1,297	1,249	1,182	1,218	1,187
OA	Total number of subjects (n)	0.14	0.15	0.21	0.25	0.25	0.28	0.30	0.29	0.25	0.25	0.24	0.23	0.23	0.23
	number of occurrences(n)	480,115	471,352	460,988	449,016	437,187	427,068	418,473	409,498	401,127	391,936	382,584	373,363	364,533	353,953
	Incidence rate (%)	15,060	15,614	15,520	13,702	12,737	12,280	12,424	12,433	12,669	12,846	12,774	12,640	13,034	12,644

**COPD**	Total number of subjects (n)	3.14	3.31	3.37	3.05	2.91	2.88	2.97	3.04	3.16	3.28	3.34	3.39	3.58	3.57
	number of occurrences(n)	498,509	502,508	505,741	508,669	508,480	508,616	509,279	510,025	511,021	511,172	512,034	512,950	513,777	513,751
	Incidence rate (%)	1,890	2,036	2,265	2,101	1,956	1,831	1,698	1,717	1,703	1,442	1,380	1,237	1,311	1,136
**CHF**	Total number of subjects (n)	0.38	0.41	0.45	0.41	0.38	0.36	0.33	0.34	0.33	0.28	0.27	0.24	0.26	0.22
	number of occurrences(n)	500,544	505,967	510,616	515,645	516,724	518,121	519,984	521,566	523,354	524,460	525,915	527,402	527,971	528,608
	Incidence rate (%)	356	407	436	538	410	384	423	470	478	458	519	575	307	328
**CKD**	Total number of subjects (n)	0.07	0.08	0.09	0.10	0.08	0.07	0.08	0.09	0.09	0.09	0.10	0.11	0.06	0.06
	number of occurrences(n)	500,301	505,790	510,289	515,204	516,452	517,771	519,480	521,046	522,602	523,504	524,619	525,975	526,902	527,095
	Incidence rate (%)	373	499	459	472	486	502	514	679	697	695	643	690	839	898
**Cataract**	Total number of subjects (n)	0.07	0.10	0.09	0.09	0.09	0.10	0.10	0.13	0.13	0.13	0.12	0.13	0.16	0.17
	number of occurrences(n)	496,995	499,585	501,461	503,197	501,881	500,459	499,803	498,815	497,370	495,959	495,120	494,146	492,767	490,593
	Incidence rate (%)	3,260	3,404	3,586	3,549	3,655	3,624	3,848	4,097	3,791	3,774	3,963	4,081	4,328	4,560
**AMD**	Total number of subjects (n)	0.66	0.68	0.72	0.71	0.73	0.72	0.77	0.82	0.76	0.76	0.80	0.83	0.88	0.93
	number of occurrences(n)	500,602	505,770	509,966	514,694	515,586	516,528	517,821	518,991	520,364	521,119	522,217	523,924	524,191	523,943
	Incidence rate (%)	664	763	697	755	782	795	729	757	743	704	703	1,230	1,299	1,429
**Hearing loss**	Total number of subjects (n)	0.13	0.15	0.14	0.15	0.15	0.15	0.14	0.15	0.14	0.14	0.13	0.23	0.25	0.27
	number of occurrences(n)	498,708	502,052	504,356	506,363	504,845	503,512	502,467	500,941	499,581	497,619	495,962	494,818	492,785	490,426
	Incidence rate (%)	2,734	3,062	3,326	3,331	3,378	3,508	3,670	3,785	3,741	3,700	3,668	3,974	4,047	4,481
**PD**	Total number of subjects (n)	0.55	0.61	0.66	0.66	0.67	0.70	0.73	0.76	0.75	0.74	0.74	0.80	0.82	0.91
	number of occurrences(n)	500,800	506,479	511,366	516,538	517,947	519,395	521,251	522,842	524,753	525,909	527,367	529,017	529,776	530,238
	Incidence rate (%)	112	175	213	234	260	271	276	289	367	350	366	513	422	444
