# Supplementary material for: Trend of incidence rate of age-related diseases: results from the National Health Insurance Service–National Sample Cohort (NHIS-NSC) database in Korea: a cross- sectional study
Source: BMC Geriatr. 2023 Dec 12;23:840. doi: 10.1186/s12877-023-04578-7 (PMC10714524; doi:10.1186/s12877-023-04578-7)
Supplement: Supplementary file 3 — Additional file 3: Supplementary Table 3. Incidence rate of age-related diseases of Female by year. [file 12877_2023_4578_MOESM3_ESM.pdf]

Supplementary Table 3. Incidence rate of age-related diseases of Female by year

		2006	2007	2008	2009	2010	2011	2012	2013	2014	2015	2016	2017	2018	2019
**HTN**	Total number of subjects (n)	2004	2005	2006	2007	2008	2009	2010	2011	2012	2013	2014	2015	2016	2017
	number of occurrences(n)	465,151	461,879	453,517	446,971	441,683	437,108	432,946	429,348	427,697	425,672	424,127	423,504	423,122	420,590
	Incidence rate (%)	10,007	11,492	9,849	8,073	7,519	7,191	6,445	5,508	5,361	4,965	4,568	4,611	5,330	5,091
**DM**	Total number of subjects (n)	2.15	2.49	2.17	1.81	1.70	1.65	1.49	1.28	1.25	1.17	1.08	1.09	1.26	1.21
	number of occurrences(n)	485,481	486,922	482,423	480,648	477,445	474,446	472,125	470,225	468,321	466,516	464,531	462,761	461,092	458,260
	Incidence rate (%)	5,760	7,719	6,215	5,568	5,335	5,129	4,917	4,970	4,843	4,907	4,768	4,639	4,922	4,802
**DL**	Total number of subjects (n)	1.19	1.59	1.29	1.16	1.12	1.08	1.04	1.06	1.03	1.05	1.03	1.00	1.07	1.05
	number of occurrences(n)	492,074	492,070	489,404	486,194	479,243	472,743	464,222	456,337	447,900	439,647	432,027	423,667	416,467	405,800
	Incidence rate (%)	6,732	8,190	8,901	9,526	9,867	11,052	10,841	11,123	10,876	10,826	11,195	11,020	12,233	11,635
**CVD**	Total number of subjects (n)	1.37	1.66	1.82	1.96	2.06	2.34	2.34	2.44	2.43	2.46	2.59	2.60	2.94	2.87
	number of occurrences(n)	496,369	498,967	500,646	502,024	500,302	498,120	496,869	495,826	495,061	494,449	494,091	494,050	493,592	492,773
	Incidence rate (%)	3,079	3,538	4,047	4,384	4,454	4,117	3,894	3,755	3,440	3,313	3,191	3,184	3,197	3,366
**IHD**	Total number of subjects (n)	0.62	0.71	0.81	0.87	0.89	0.83	0.78	0.76	0.69	0.67	0.65	0.64	0.65	0.68
	number of occurrences(n)	493,261	495,055	494,909	495,397	492,933	491,119	490,048	489,012	489,012	488,552	488,472	488,059	488,092	487,399
	Incidence rate (%)	4,044	4,666	4,481	4,375	3,886	3,648	3,373	2,968	2,964	2,827	2,820	2,402	2,501	2,389
**Osteoporosis**	Total number of subjects (n)	0.82	0.94	0.91	0.88	0.79	0.74	0.69	0.61	0.61	0.58	0.58	0.49	0.51	0.49
	number of occurrences(n)	486,820	482,259	480,800	477,252	471,288	465,932	461,104	456,246	452,481	449,113	445,860	444,052	441,998	439,708
	Incidence rate (%)	8,526	7,282	8,361	8,091	7,718	7,577	7,237	6,593	6,099	5,763	5,043	4,953	4,946	5,150
**OA**	Total number of subjects (n)	1.75	1.51	1.74	1.70	1.64	1.63	1.57	1.45	1.35	1.28	1.13	1.12	1.12	1.17
	number of occurrences(n)	451,010	433,081	413,658	394,515	378,618	365,873	355,843	346,415	337,694	328,676	320,811	312,591	305,491	296,678
	Incidence rate (%)	22,869	22,513	20,641	16,996	14,743	13,410	12,946	12,584	12,370	11,795	11,727	11,165	11,426	10,814

**COPD**	Total number of subjects (n)	5.07	5.20	4.99	4.31	3.89	3.67	3.64	3.63	3.66	3.59	3.66	3.57	3.74	3.65
	number of occurrences(n)	496,960	500,432	503,234	506,046	506,478	506,554	507,198	508,254	509,357	509,896	511,124	512,604	513,458	514,021
	Incidence rate (%)	1,848	1,951	2,052	1,829	1,869	1,696	1,466	1,527	1,430	1,081	980	965	839	751
**CHF**	Total number of subjects (n)	0.37	0.39	0.41	0.36	0.37	0.33	0.29	0.30	0.28	0.21	0.19	0.19	0.16	0.15
	number of occurrences(n)	498,682	503,302	507,353	511,685	512,737	514,033	515,869	517,406	519,421	520,956	522,569	524,344	525,401	526,196
	Incidence rate (%)	659	715	831	910	732	708	729	614	630	614	590	580	449	407
**CKD**	Total number of subjects (n)	0.13	0.14	0.16	0.18	0.14	0.14	0.14	0.12	0.12	0.12	0.11	0.11	0.09	0.08
	number of occurrences(n)	498,917	503,802	508,034	512,765	514,291	515,739	517,626	519,542	521,404	522,958	524,525	526,325	527,459	528,000
	Incidence rate (%)	373	409	407	463	440	432	400	515	466	484	445	513	585	578
**Cataract**	Total number of subjects (n)	0.07	0.08	0.08	0.09	0.09	0.08	0.08	0.10	0.09	0.09	0.08	0.10	0.11	0.11
	number of occurrences(n)	491,774	492,219	492,060	490,941	488,421	485,617	483,721	482,118	479,717	477,866	476,424	474,650	473,006	470,147
	Incidence rate (%)	5,026	5,252	5,638	5,070	5,112	4,931	4,935	5,272	4,849	4,812	5,039	5,047	5,558	5,788
**AMD**	Total number of subjects (n)	1.02	1.07	1.15	1.03	1.05	1.02	1.02	1.09	1.01	1.01	1.06	1.06	1.18	1.23
	number of occurrences(n)	498,935	503,482	507,149	511,452	512,595	513,266	514,410	515,760	517,029	517,944	518,977	520,687	521,106	520,636
	Incidence rate (%)	803	927	869	961	1,111	1,068	952	1,100	1,059	1,035	1,004	1,522	1,826	1,989
**Hearing loss**	Total number of subjects (n)	0.16	0.18	0.17	0.19	0.22	0.21	0.19	0.21	0.20	0.20	0.19	0.29	0.35	0.38
	number of occurrences(n)	496,720	499,215	500,892	502,073	500,292	498,471	497,009	494,959	493,110	490,856	488,523	487,007	484,473	481,037
	Incidence rate (%)	3,112	3,468	3,896	3,925	3,976	4,159	4,438	4,477	4,510	4,526	4,367	4,768	4,992	5,082
**PD**	Total number of subjects (n)	0.63	0.69	0.78	0.78	0.79	0.83	0.89	0.90	0.91	0.92	0.89	0.98	1.03	1.06
	number of occurrences(n)	499,304	504,388	508,865	513,665	515,292	516,802	518,624	520,509	522,602	524,004	525,606	527,514	528,329	528,966
	Incidence rate (%)	191	246	323	364	380	429	357	433	532	462	447	633	496	507
