# Supplementary material for: Trend of incidence rate of age-related diseases: results from the National Health Insurance Service–National Sample Cohort (NHIS-NSC) database in Korea: a cross- sectional study
Source: BMC Geriatr. 2023 Dec 12;23:840. doi: 10.1186/s12877-023-04578-7 (PMC10714524; doi:10.1186/s12877-023-04578-7)
Supplement: Supplementary file 4 — Additional file 4: Supplementary Table 4. Incidence rate of age-related diseases of Male by age group. [file 12877_2023_4578_MOESM4_ESM.pdf]

Supplementary Table 4 Incidence rate of age-related diseases of Male by age group

		0	10	20	30	40	50	60	70	80	90	Total
**HTN**	Total number of subjects (n)	44,515	53,136	72,801	69,847	71,960	58,677	29,518	9,929	2,617	248	413,248
	number of occurrences(n)	13	194	758	2,066	3,972	4,648	2,982	1,174	294	40	16,141
	Incidence rate (%)	0.03	0.37	1.04	2.96	5.52	7.92	10.10	11.82	11.23	16.13	3.91
**DM**	Total number of subjects (n)	44,538	52,962	72,983	72,009	77,839	68,998	40,510	17,276	6,032	630	453,777
	number of occurrences(n)	35	238	396	1,228	2,738	3,340	2,652	1,324	376	22	12,349
	Incidence rate (%)	0.08	0.45	0.54	1.71	3.52	4.84	6.55	7.66	6.23	3.49	2.72
**DL**	Total number of subjects (n)	44,454	52,215	70,381	65,050	64,377	54,085	30,207	13,635	5,448	626	400,478
	number of occurrences(n)	130	802	1,662	3,806	5,854	6,168	4,234	1,922	634	42	25,254
	Incidence rate (%)	0.29	1.54	2.36	5.85	9.09	11.40	14.02	14.10	11.64	6.71	6.31
**CVD**	Total number of subjects (n)	44,518	53,196	74,104	74,126	84,652	82,376	53,748	24,334	7,370	657	499,081
	number of occurrences(n)	20	48	108	266	720	1,648	2,004	1,590	654	64	7,122
	Incidence rate (%)	0.04	0.09	0.15	0.36	0.85	2.00	3.73	6.53	8.87	9.74	1.43
**IHD**	Total number of subjects (n)	44,558	53,246	73,797	73,111	82,164	78,003	49,495	23,021	7,777	774	485,946
	number of occurrences(n)	1	42	182	398	812	1,450	1,594	1,024	368	52	5,923
	Incidence rate (%)	0.00	0.08	0.25	0.54	0.99	1.86	3.22	4.45	4.73	6.72	1.22
**Osteoporosis**	Total number of subjects (n)	44,578	53,341	74,408	74,812	86,280	86,811	60,129	29,170	9,193	811	519,533
	number of occurrences(n)	10	18	50	56	196	436	588	1,016	362	32	2,764
	Incidence rate (%)	0.02	0.03	0.07	0.07	0.23	0.50	0.98	3.48	3.94	3.95	0.53
**OA**	Total number of subjects (n)	44,117	49,165	59,309	52,288	53,971	43,913	21,599	7,011	1,742	134	333,249
	number of occurrences(n)	338	2,002	3,918	4,364	5,216	5,164	3,206	1,076	232	14	25,530
	Incidence rate (%)	0.77	4.07	6.61	8.35	9.66	11.76	14.84	15.35	13.32	10.45	7.66

**COPD**	Total number of subjects (n)	44,481	53,079	74,045	74,358	85,536	85,641	58,282	27,997	9,013	825	513,257
	number of occurrences(n)	16	10	46	70	158	402	742	752	266	28	2,490
	Incidence rate (%)	0.04	0.02	0.06	0.09	0.18	0.47	1.27	2.69	2.95	3.39	0.49
**CHF**	Total number of subjects (n)	44,570	53,349	74,485	74,945	86,644	87,611	61,784	32,373	11,698	1,134	528,593
	number of occurrences(n)	1	4	14	16	58	96	180	214	154	34	771
	Incidence rate (%)	0.00	0.01	0.02	0.02	0.07	0.11	0.29	0.66	1.32	3.00	0.15
**CKD**	Total number of subjects (n)	44,576	53,351	74,411	74,796	86,281	87,102	61,249	31,847	11,455	1,082	526,150
	number of occurrences(n)	9	20	36	80	168	320	478	600	354	24	2,089
	Incidence rate (%)	0.02	0.04	0.05	0.11	0.19	0.37	0.78	1.88	3.09	2.22	0.40
**Cataract**	Total number of subjects (n)	44,563	53,223	74,174	74,614	85,346	82,449	50,236	16,505	3,636	299	485,045
	number of occurrences(n)	2	14	44	114	656	2,468	4,032	2,496	522	24	10,372
	Incidence rate (%)	0.00	0.03	0.06	0.15	0.77	2.99	8.03	15.12	14.36	8.03	2.14
**AMD**	Total number of subjects (n)	44,583	53,315	74,247	74,749	86,174	86,450	60,272	30,559	10,673	998	522,020
	number of occurrences(n)	4	4	16	68	226	610	1,174	1,152	410	26	3,690
	Incidence rate (%)	0.01	0.01	0.02	0.09	0.26	0.71	1.95	3.77	3.84	2.61	0.71
**Hearing loss**	Total number of subjects (n)	43,649	50,551	69,631	70,178	80,505	79,993	53,899	25,420	8,483	741	483,050
	number of occurrences(n)	280	526	756	990	1,166	1,588	1,874	1,412	534	58	9,184
	Incidence rate (%)	0.64	1.04	1.09	1.41	1.45	1.99	3.48	5.55	6.29	7.83	1.90
**PD**	Total number of subjects (n)	44,585	53,332	74,386	74,872	86,601	87,810	62,341	32,874	11,875	1,168	529,844
	number of occurrences(n)	10	36	24	36	54	110	152	300	152	8	882
	Incidence rate (%)	0.02	0.07	0.03	0.05	0.06	0.13	0.24	0.91	1.28	0.68	0.17
