# Supplementary material for: Trend of incidence rate of age-related diseases: results from the National Health Insurance Service–National Sample Cohort (NHIS-NSC) database in Korea: a cross- sectional study
Source: BMC Geriatr. 2023 Dec 12;23:840. doi: 10.1186/s12877-023-04578-7 (PMC10714524; doi:10.1186/s12877-023-04578-7)
Supplement: Supplementary file 5 — Additional file 5: Supplementary Table 5. Incidence rate of age-related diseases of Female by age group. [file 12877_2023_4578_MOESM5_ESM.pdf]

Supplementary Table 5 Incidence rate of age-related diseases of Female by age group

		0	10	20	30	40	50	60	70	80	90	Total
**HTN**	Total number of subjects (n)	42,271	49,421	65,975	67,438	76,329	65,182	34,238	11,076	3,334	565	415,829
	number of occurrences(n)	16	94	276	732	2,218	3,406	2,884	1,434	460	60	11,580
	Incidence rate (%)	0.04	0.19	0.42	1.09	2.91	5.23	8.42	12.95	13.80	10.62	2.78
**DM**	Total number of subjects (n)	42,286	49,178	65,551	66,716	77,060	73,053	44,757	20,847	10,670	2,331	452,449
	number of occurrences(n)	24	106	370	770	1,476	3,002	2,682	1,534	682	104	10,750
	Incidence rate (%)	0.06	0.22	0.56	1.15	1.92	4.11	5.99	7.36	6.39	4.46	2.38
**DL**	Total number of subjects (n)	42,268	48,335	63,247	61,949	68,484	53,950	25,284	12,401	8,315	2,244	386,477
	number of occurrences(n)	290	536	1,662	2,702	4,854	7,512	3,984	1,988	942	110	24,580
	Incidence rate (%)	0.69	1.11	2.63	4.36	7.09	13.92	15.76	16.03	11.33	4.90	6.36
**CVD**	Total number of subjects (n)	42,271	49,490	66,272	68,947	81,741	81,660	55,600	28,537	12,989	2,281	489,788
	number of occurrences(n)	14	26	106	246	564	1,652	2,040	1,882	1,132	188	7,850
	Incidence rate (%)	0.03	0.05	0.16	0.36	0.69	2.02	3.67	6.59	8.72	8.24	1.60
**IHD**	Total number of subjects (n)	42,291	49,506	66,208	68,494	80,969	79,828	53,209	27,811	14,145	2,726	485,187
	number of occurrences(n)	5	46	128	200	424	952	1,238	1,054	514	84	4,645
	Incidence rate (%)	0.01	0.09	0.19	0.29	0.52	1.19	2.33	3.79	3.63	3.08	0.96
**Osteoporosis**	Total number of subjects (n)	42,308	49,573	66,491	68,620	80,491	72,042	35,639	11,780	5,560	1,378	433,882
	number of occurrences(n)	12	18	82	288	962	4,360	3,806	1,416	558	78	11,580
	Incidence rate (%)	0.03	0.04	0.12	0.42	1.20	6.05	10.68	12.02	10.04	5.66	2.67
**OA**	Total number of subjects (n)	41,874	46,448	53,843	48,172	47,689	28,896	9,901	2,764	948	235	280,770
	number of occurrences(n)	253	1,752	3,596	3,754	5,268	4,980	1,880	550	166	36	22,235
	Incidence rate (%)	0.60	3.77	6.68	7.79	11.05	17.23	18.99	19.90	17.51	15.32	7.92

**COPD**	Total number of subjects (n)	42,248	49,417	66,288	69,008	82,488	84,957	61,913	36,020	18,651	3,267	514,257
	number of occurrences(n)	13	24	50	58	118	226	328	354	272	42	1,485
	Incidence rate (%)	0.03	0.05	0.08	0.08	0.14	0.27	0.53	0.98	1.46	1.29	0.29
**CHF**	Total number of subjects (n)	42,297	49,578	66,656	69,684	83,599	86,796	64,666	38,924	20,769	3,715	526,684
	number of occurrences(n)	4	-	6	18	26	88	142	274	228	68	854
	Incidence rate (%)	0.01	-	0.01	0.03	0.03	0.10	0.22	0.70	1.10	1.83	0.16
**CKD**	Total number of subjects (n)	42,312	49,592	66,637	69,594	83,428	86,514	64,553	39,342	21,830	4,056	527,858
	number of occurrences(n)	5	12	22	40	62	140	284	416	380	56	1,417
	Incidence rate (%)	0.01	0.02	0.03	0.06	0.07	0.16	0.44	1.06	1.74	1.38	0.27
**Cataract**	Total number of subjects (n)	42,308	49,487	66,423	69,361	82,713	82,121	49,702	15,396	4,922	1,096	463,529
	number of occurrences(n)	12	14	30	88	728	3,638	5,478	2,722	776	42	13,528
	Incidence rate (%)	0.03	0.03	0.05	0.13	0.88	4.43	11.02	17.68	15.77	3.83	2.92
**AMD**	Total number of subjects (n)	42,307	49,551	66,431	69,441	83,244	85,868	62,564	35,890	19,229	3,593	518,118
	number of occurrences(n)	-	8	18	74	240	880	1,652	1,414	610	58	4,954
	Incidence rate (%)	-	0.02	0.03	0.11	0.29	1.02	2.64	3.94	3.17	1.61	0.96
**Hearing loss**	Total number of subjects (n)	41,644	47,371	61,798	63,804	76,306	77,473	54,825	30,670	15,879	2,905	472,675
	number of occurrences(n)	264	514	1,082	1,218	1,348	1,962	1,928	1,626	846	88	10,876
	Incidence rate (%)	0.63	1.09	1.75	1.91	1.77	2.53	3.52	5.30	5.33	3.03	2.30
**PD**	Total number of subjects (n)	42,313	49,581	66,581	69,587	83,532	86,833	65,109	39,642	21,650	4,027	528,855
	number of occurrences(n)	-	8	42	42	42	102	178	294	192	22	922
	Incidence rate (%)	-	0.02	0.06	0.06	0.05	0.12	0.27	0.74	0.89	0.55	0.17
