# Supplementary material for: Trend of incidence rate of age-related diseases: results from the National Health Insurance Service–National Sample Cohort (NHIS-NSC) database in Korea: a cross- sectional study
Source: BMC Geriatr. 2023 Dec 12;23:840. doi: 10.1186/s12877-023-04578-7 (PMC10714524; doi:10.1186/s12877-023-04578-7)

Supplementary Figure 2. Incidence rate of age-related diseases of Male by age group per 100,000 persons

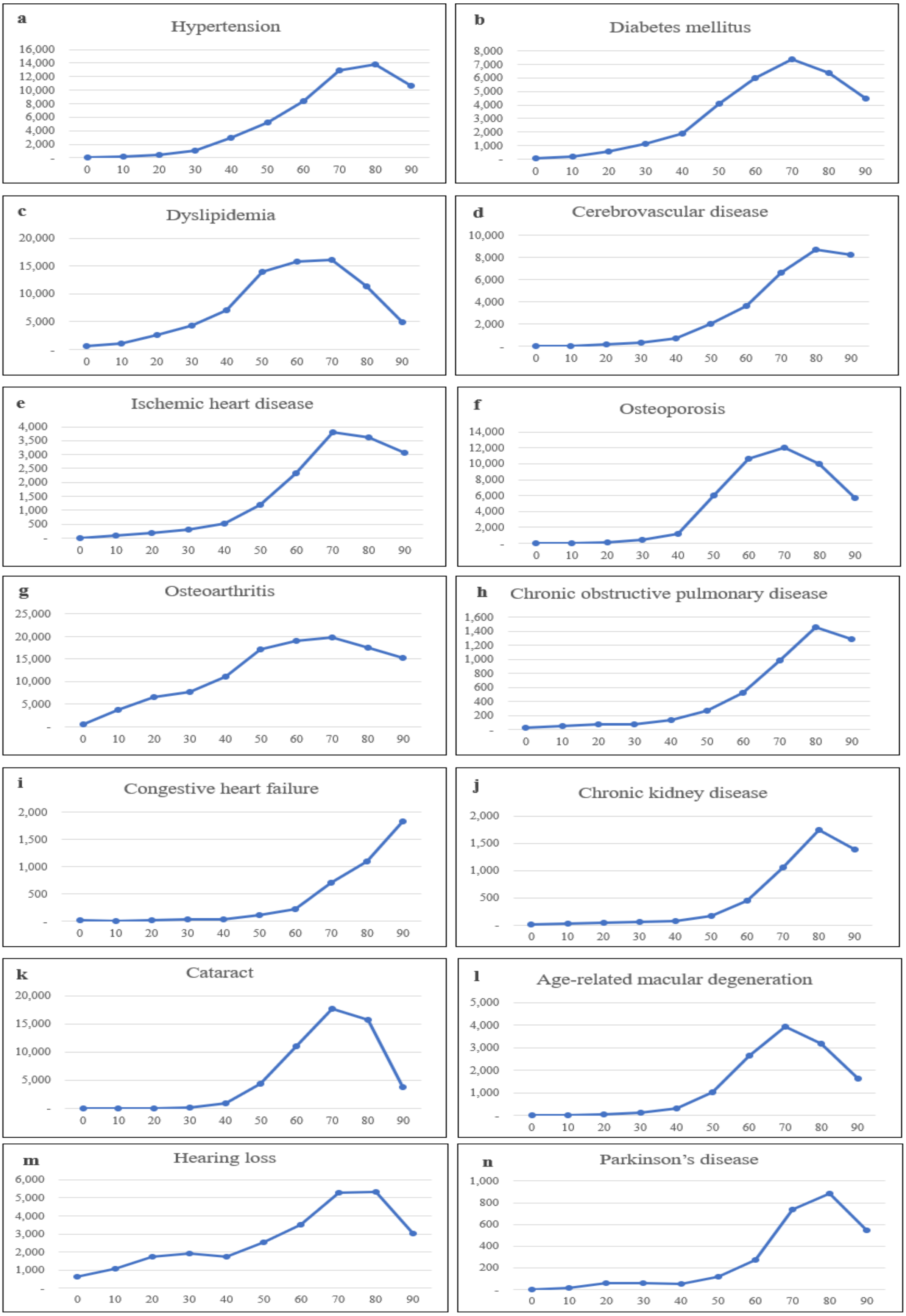

Supplement: Supplementary file 7 — Additional file 7: Supplementary Figure 2. Incidence rate of age-related diseases of Male by age group per 100,000 persons. [file 12877_2023_4578_MOESM7_ESM.pdf]
